# The separation of several organophosphate pesticides on immobilized polysaccharide chiral stationary phases

**DOI:** 10.1002/chir.23473

**Published:** 2022-05-31

**Authors:** William L. Champion, William L. Watts, Weston J. Umstead

**Affiliations:** ^1^ Senior Method Development Chemist, WLC, WLW, Champion Watts Technology and Method Development Chiral Technologies, Inc West Chester Pennsylvania USA; ^2^ Technology and Business Development Manager, WJU Umstead Technology and Method Development Chiral Technologies, Inc West Chester Pennsylvania USA

**Keywords:** chiral HPLC, normal phase HPLC, organo‐phosphate pesticides, polysaccharides

## Abstract

While not initially a focus or priority, in recent decades, an emphasis has been placed on the activity of individual enantiomers of widely used pesticides. Of particular note are organophosphorus‐based pesticides like fenamiphos and profenofos, as examples. This work explores the enantioselective high‐performance liquid chromatography (HPLC) separations of seven such organophosphorus pesticides (OP's) on the library of immobilized polysaccharide‐based chiral stationary phases (CSPs) with normal phase hexane/alcohol mixtures. Further exploration of the effect of mobile phase strength and temperature on several of the separations was performed using simple factorial design. Equivalent retention of the first eluting enantiomer of several combinations of temperature and mobile phase was compared for peak shape, selectivity, and resolution. Similarly, equivalent selectivity of several combinations of temperature and mobile phase was compared for peak shape, retention of the first eluting enantiomer, and resolution. The results of this study make available several new chiral separations of the OPs included in the work that were not previously documented, including separations on the three most recently commercialized phases, Chiralpak IH, IJ, and IK. Additionally, sufficient understanding was obtained to be able to predict the trade‐off of resolution, analysis time, peak sharpness (and thus improve limit‐of‐detection [LOD]/limit‐of‐quantification [LOQ]), robustness, and convenience of conditions for further application optimization.

## Graphical abstract

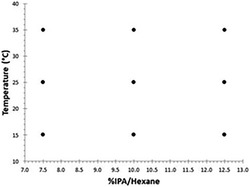

## 1 INTRODUCTION

The investigation and study of the activity and uses of pesticides can be traced back more than 4500 years to the first uses of sulfur‐containing compounds by the Sumerians to help ward off insects and other deleterious plant pests^1^ Over the millennia, as science and technology have advanced, so too have the pesticides and herbicides we use, shifting from things naturally extracted or occurring, to those synthetically manufactured. Many commercial chiral pesticides are often sold as racemates, containing an equal percentage of both “left‐handed” and “right‐handed” enantiomers. Much like for pharmaceuticals, the intended biological activity or the environmental fate may be different for the individual enantiomers. With regulatory changes in the EU aimed at ensuring the use of pesticides equates to safety for human exposure,^2^ more pronounced focus has been placed on the effects of the individual enantiomers of said pesticides (where applicable).

**FIGURE 1:**
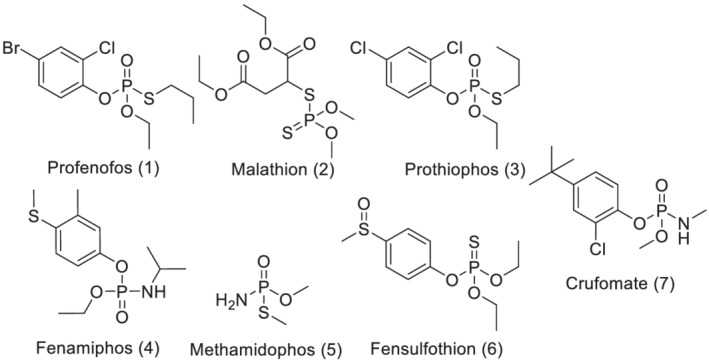
Organophosphate pesticides

The requirements for an element to be considered a chiral center are straightforward—it must have a tetrahedral geometry or more simply put be able to form four bonds with other elements, including lone pair of electrons, and those four groups must all be different. Carbon is the most commonly occurring and identified chiral center; however, nitrogen, phosphorus, and sulfur can also form tetrahedral geometries and can therefore also be chiral centers. As shown in Figure 1, all seven of the compounds in this study have a single chiral center. Five have phosphate or thiophosphate as their chiral center. For Malathion (Compound 2), the chiral center is a carbon, and for fensulfothion (Compound 6), the chiral center is a sulfoxide. Note that for both Malathion and fensulfothion, the phosphate is symmetrical (i.e., achiral).

This work is a follow‐up to previously developed enantioselective analyses of chiral organophosphate pesticides (OPs).^3^ In the almost 20 years since its publication, the technology of enantioselective separations has evolved. There are new, unique chiral selectors not available at that time, as well as smaller particle sizes like 5, 3, and sub‐2 μm. While some of these new technologies have been explored in part,^4^, ^5^, ^6^, ^7^, ^8^, ^9^, ^10^, ^11^, ^12^, ^13^ a recent assessment with a complete compilation of immobilized polysaccharide‐based chiral stationary phases (CSPs) is not presently available.

Immobilized CSPs offer several advantages over traditional coated CSPs.^14^, ^15^, ^16^, ^17^, ^18^, ^19^, ^20^, ^21^, ^22^, ^23^, ^24^, ^25^, ^26^ Because these columns are stable to a wider range of liquid chromatography (LC) mobile phase solvents, there is an expanded opportunity to find a separation from the greater ability to control the separation (via changing of elution strength and/or solvation interactions with the CSP and analyte. The immobilization process has provided access to chiral selectors that were previously described but not stable as coated phases,^27^ further increasing the likelihood of finding a separation.^15^ This publication provides several improved separations of the pesticides shown in Figure 1, including improvements in resolution, selectivity, and a reduction in analysis time. As an example, the separation of Profenofos (1) was previously reported on Chiralpak AD using a mobile phase of Heptane‐EtOH = 98–2 (v/v)^3^; the unique selectivity of Chiralpak IC as reported in this publication afforded a more convenient ratio of 90–10 (v/v) with an improved resolution (2.6 vs. 1.2).

Although immobilization offers an increased access to more mobile phase solvents, alkane/alcohol mobile phases were used for this work for several reasons: (1) to allow for a direct comparison to previous works; (2) the relative ease of use, compared with say halogenated solvents which require more stringent waste disposal; and (3) the low wavelength UV‐cutoffs for hexane, EtOH, and IPA allows for lower analysis wavelengths, and thus, the limit‐of‐detection (LOD) or limit‐of‐quantification (LOQ) obtained are lower should these values need to be determined. While alkane/alcohol mobile phases are not typically used with LC/MS applications, they are desirable for preparative separations and can be used for in‐process control for enatio‐selective synthesis or purification or for release analysis of formulated product.

A systematic approach (e.g., automated column screening and simple experimental design) was used to speed up and simplify the evaluation, resulting in a more efficient optimization process. There is a brief discussion of the difficulty of quantitative measurement of resolution of peaks of significantly different sizes (e.g., 1000‐fold or greater) and examples of the variable effect of temperature on selectivity in chiral separations.

## 2 MATERIALS AND METHODS

### 2.1 System 1

The high‐performance liquid chromatography (HPLC) instrument used in initial column screening was an Agilent 1200 configured with low‐pressure mixing, quaternary mobile phase delivery system, vacuum degasser, autosampler, photodiode array UV detector, 12 port Valco column switching valve, and PDR‐Chiral in‐line polarimeter (optical rotation is obtained at 675 nm). Column temperature was not controlled. The instrument was controlled by an Agilent ChemStation Version B‐04.03.^16^

### 2.2 System 2

The HPLC instrument used for further investigation and optimization of separations was an Agilent 1200 configured with high‐pressure mixing binary mobile phase delivery system, vacuum degasser, autosampler, temperature controlled column compartment, and photodiode array UV detector. The instrument was controlled by an Agilent ChemStation Version B‐04.03.^16^

### 2.3 Solvents

The mobile phase was mixed by the solvent delivery system. The hexanes used were purchased from Scientific Equipment Company (Aston, PA, USA) and contained 95% n‐hexane. The isopropanol (IPA) and ethanol (EtOH) were also purchased from Scientific Equipment Company, the latter supplied as denatured Reagent Alcohol (90% EtOH, 5% MeOH, and 5% isopropanol (IPA) v/v/v).

### 2.4 Pesticide standards

The pesticide standard for Crufomate was obtained from ChemServices in West Chester, PA, USA. The other standards were obtained from Sigma Aldrich (USA).

### 2.5 CSPs

The chiral columns used were Chiralpak IA‐3 [amylose tris (3,5‐dimethylphenylcarbamate)], Chiralpak IB‐N‐3 [cellulose tris (3,5‐dimethylphenylcarbamate)], Chiralpak IC‐3 [cellulose tris (3,5‐dichlorophenylcarbamate)], Chiralpak ID‐3 [amylose tris (3‐chlorophenylcarbamate)], Chiralpak IE‐3 [amylose tris (3,5‐dichlorophenylcarbamate)], Chiralpak IF‐3 [amylose tris (3‐chloro‐4‐methylphenylcarbamate)], Chiralpak IG‐3 [amylose tris (3‐chloro‐5‐methylphenylcarbamate)], Chiralpak IH‐3 [amylose tris (S)‐α‐methylbenzylcarbamate)], Chiralpak IJ‐3 [cellulose tris (4‐ methylbenzoate)], and Chiralpak IK [cellulose tris (3‐chloro‐5‐methylphenylcarbamate)]. All columns were 4.6 mm inner diameter (i.d.) × 150 mm length. The particle size was nominal 3 μm immobilized on spherical silica gel, except for the Chiralpak IK, which was 5 μm immobilized on spherical silica gel.

### 2.6 Determination of retention and calculation of retention factor

Retention factor (k) was determined using a nominal unretained solvent volume or “void volume” of 1.6 ml for the 150 mm length columns. A flow rate of 1 ml/min was used so retention times (t_R_) can be directly compared. t_R‐1_ and k_−1_ refer to first eluting enantiomer and t_R‐2_ and k_−2_ refer to the second eluting enantiomer.

### 2.7 Quality of separation

Separation was measured qualitatively as separation between the enantiomer peaks at baseline rather than as resolution. In achiral chromatography (e.g., RPLC and NPLC), closely eluting components typically have similar retention mechanisms, so their peak widths are often similar. In chiral chromatography, the peak of the more retained enantiomer sometimes shows significantly more tailing (and thus greater peak width) than the earlier eluting enantiomer. The amount of peak tailing for the more retained enantiomer has little effect on the separation but greatly affects the calculated resolution and thus typically under measures the quality of the separation.^28^ As the difference in relative size of the peaks increases, it becomes increasingly more difficult to reliably measure the area (or height) of the smaller peak if the peaks are not at least baseline resolved.^28^, ^29^, ^30^, ^31^ In chiral chromatography, the objective is often the accurate measurement of the low‐level, undesired enantiomer. The peak area of the undesired enantiomer can often be 0.1% (or less) relative to the desired enantiomer, so there is a premium placed on complete separation of the enantiomer peaks.

Rather than measuring resolution based upon peak width at 4σ,^32^ the separations obtained in screening were graded qualitatively based upon space between the peaks:

- Co‐eluting (C)—possible peak broadening but single retention time.
- Split (S)—separate retention times, but peaks separated at <50% of peak height.
- Partial (P)—peaks separated at between ~50% of peak height and ~5% of peak height.
- Less than baseline resolved (<B)—almost completely resolved but not truly baseline resolved.
- Baseline resolved (B)—truly baseline resolved but just barely. A slight decrease in selectivity or increase in peak width could cause loss of complete separation.
- Well resolved (>B)—better than baseline separation. A small decrease in selectivity or small increase in peak width would not compromise the separation. This would generally be considered an ideal separation.
- Resolution unnecessarily large (> > B)—separation is so large that retention time of the later eluting enantiomer unnecessarily increases the run time and probably unnecessarily increases the width of the peak of the later eluting enantiomer.

## 3 RESULTS AND DISCUSSION

### 3.1 Screening

All seven compounds were screened at ambient temperature using hexane/EtOH and hexane/IPA = 80/20, 90/10, and 95/5 on Chiralpak IA‐3, IB‐N‐3, IC‐3, ID‐3, IE‐3, IF‐3, IG‐3, IH‐3, IJ‐3, and IK‐5. This was accomplished using the library of columns connected to the HPLC by a switching valve and automated for unattended screening. The seven compounds were screened on all of the CSPs and mobile phases first, and optimization was performed afterward. Baseline separations or better were found on at least one of these columns for all of the compounds, except prothiophos. For several of the compounds, good separation was found on several columns. (Prothiophos was specifically included in this study because good separation conditions had not been obtained in the previous work^3^ using alkane/alcohol mobile phase).

Table 1 shows screening results (typically at 10% alcohol). If there was appreciable improvement in the separation at 5% or 20% alcohol, those results are also shown. To simplify the presentation, only separations that showed promise (i.e., on screening, slightly less than baseline separation or better was obtained) are shown.

**TABLE 1:** Summary of results of screening

MP	Column	Cmp	OE	Sepn 20%	k(1)	k(2)	Sepn 10%	k(1)	k(2)	Sepn 5%	k(1)	k(2)
**IPA**	IC	**1**	**+/−**	‐‐‐	‐‐‐	‐‐‐	B	2.7	3.1	>B	3.4	4.6
**IPA**	IG	**1**	**+/−**	‐‐‐	‐‐‐	‐‐‐	P	3.4	3.8	<B	5.9	6.6
**EtOH**	IK	**1**	**+/−**	‐‐‐	‐‐‐	‐‐‐	<B	1.2	1.4	‐‐‐	‐‐‐	‐‐‐
**IPA**	IK	**1**	**+/−**	‐‐‐	‐‐‐	‐‐‐	>B	1.7	2.4	‐‐‐	‐‐‐	‐‐‐
**EtOH**	IC	**2**	**−/+**	<B	1.3	1.5	B	2.0	2.3	‐‐‐‐	‐‐‐	‐‐‐
**IPA**	IC	**2**	**−/+**	P	2.8	3.1	<B	5.4	6.0	‐‐‐	‐‐‐	‐‐‐
**EtOH**	IE	**2**	**+/−**	‐‐‐	‐‐‐	‐‐‐	P	2.0	2.2	<B	3.0	3.3
**EtOH**	IG	**2**	**−/+**	P	1.5	1.8	<B	2.3	2.6	>B	3.5	4.1
**EtOH**	IK	**2**	**−/+**	‐‐‐	‐‐‐	‐‐‐	B	1.7	2.1	‐‐‐	‐‐‐	‐‐‐
**IPA**	IK	**2**	**−/+**	‐‐‐	‐‐‐	‐‐‐	>B	3.3	4.1	‐‐‐	‐‐‐	‐‐‐
**EtOH**	IA	**4**	**+/−**	<B	0.9	1.1	B	1.6	2.0	>B	3.2	3.9
**IPA**	IA	**4**	**+/−**	P	0.9	1.1	B	1.9	2.3	>B	4.1	4.9
**EtOH**	IC	**4**	**−/+**	B	1.3	1.6	>B	2.4	3.1	X	> 8	
**IPA**	IC	**4**	**−/+**	> > B	2.8	3.8	>B	6.1	> 6.5	X	> 8	
**EtOH**	ID	**4**	**+/−**	P	0.9	0.9	P	1.5	1.7	<B, t	3.0	3.4
**IPA**	ID	**4**	**+/−**	<B	1.7	1.9	>B	3.2	3.8	<B, b	6.3	7.6
**EtOH**	IE	**4**	**+/−**	<B	1.7	2.0	>B	3.3	3.9	>B, t	6.1	7.4
**IPA**	IE	**4**	**+/−**	B	2.6	3.0	>B, t	5.4	6.3	X	> 15	
**EtOH**	IF	**4**	**+/−**	P	1.1	1.3	<B	2.1	2.4	B	3.5	3.9
**IPA**	IF	**4**	**+/−**	P	1.4	1.6	<B	2.9	3.3	<B, b	6.3	6.9
**EtOH**	IG	**4**	**+/−**	>B	1.6	1.9	> > B	2.9	3.8	> > B	6.0	8.0
**EtOH**	IH	**4**	**−/+**	<B	1.4	1.9	>B	2.6	3.2	>B, b	4.8	5.6
**IPA**	IH	**4**	**−/+**	>B, t	3.7	4.6	X	> 6.5		X	> 8	
**EtOH**	IK	**4**	**−/+**	‐‐‐	‐‐‐	‐‐‐	>B	1.8	2.2	‐‐‐	‐‐‐	‐‐‐
**IPA**	IK	**4**	**−/+**	‐‐‐	‐‐‐	‐‐‐	> > B	3.4	4.8	‐‐‐	‐‐‐	‐‐‐
**EtOH**	IBN	**5**	**+/−**	B	1.3	1.6	> > B	3.3	4.1	X	> 8	
**IPA**	IBN	**5**	**+/−**	>B	2.0	2.8	> > B	5.6	> 12	X	>	
**EtOH**	IC	**5**	**+/−**	>B	3.5	4.1	>B	9.6	> 11	X	> 11	
**IPA**	IC	**5**	**+/−**	B, t,b	8.6	10.0	X	>11		X	> 11	
**EtOH**	IH	**5**	**+/−**	B	3.3	3.9	B, b	8.5	9.8	‐‐‐	‐‐‐	‐‐‐
**EtOH**	IK	**5**	**+/−**	‐‐‐	‐‐‐	‐‐‐	> > B	6.5	8.2	‐‐‐	‐‐‐	‐‐‐
**IPA**	IK	**5**	**+/−**	‐‐‐	‐‐‐	‐‐‐	X	> 8	‐‐‐	‐‐‐	‐‐‐	‐‐‐
**EtOH**	IJ	**5**	**+/−**	‐‐‐	‐‐‐	‐‐‐	<B	2.9	3.2	‐‐‐	‐‐‐	‐‐‐
**IPA**	IJ	**5**	**+/−**	‐‐‐	‐‐‐	‐‐‐	P	4.1	4.4	‐‐‐	‐‐‐	‐‐‐
**EtOH**	IA	**6**	**−/+**	‐‐‐	‐‐‐	‐‐‐	B	3.9	4.6	‐‐‐	‐‐‐	‐‐‐
**EtOH**	IF	**6**	**+/−**	‐‐‐	‐‐‐	‐‐‐	B, t	6.0	6.9	>B, t	12.0	13.3
**EtOH**	IH	**6**	**+/−**	‐‐‐	‐‐‐	‐‐‐	> > B	9.1	10.9	X	10.8	>20
**EtOH**	IA	**7**	**−/+**	‐‐‐	‐‐‐	‐‐‐	>B	1.6	2.0	‐‐‐	‐‐‐	‐‐‐
**EtOH**	IB‐N	**7**	**+/−**	‐‐‐	‐‐‐	‐‐‐	<B	1.3	1.4	‐‐‐	‐‐‐	‐‐‐
**EtOH**	IC	**7**	**−/+**	‐‐‐	‐‐‐	‐‐‐	B	2.6	3.0	‐‐‐	‐‐‐	‐‐‐
**EtOH**	IH	**7**	**−/+**	‐‐‐	‐‐‐	‐‐‐	B	2.3	2.6	‐‐‐	‐‐‐	‐‐‐
**EtOH**	AD	**7**	**−/+**	‐‐‐	‐‐‐	‐‐‐	> > B	1.8	2.8	‐‐‐	‐‐‐	‐‐‐
**EtOH**	IG	**7**	**−/+**	‐‐‐	‐‐‐	‐‐‐	> > B	2.7	3.7	‐‐‐	‐‐‐	‐‐‐

*Note*: ‐‐‐ not run. b: broad peak(s). t(0) = 1.6 min. “OE”: order of elution based upon sign of rotation at 675 nm in mobile phase. X: retn time excessively long (typically >20 min, k > 11). t: at least one of the peaks tails. P: partial separation. <B: less than baseline resolution. B: peaks barely baseline resolved. >B: better than baseline resolution. > > B: very well resolved, possibly excessive separartion (e.g., unnecessarily long analysis time).

***Profenofos (Compound 1)*** Better than baseline separation was obtained on Chiralpak IC using 5% IPA/hexane mobile phase. Less than baseline separation was obtained on Chiralpak IG using 5% IPA/hexane mobile phase. Only partial separation was obtained on Chiralpak IK using 10% EtOH/hexane mobile phase.

***Malathion (Compound 2)*** Baseline separation was obtained on Chiralpak IC using 10% EtOH/hexane and 10% IPA/hexane mobile phase, on Chiralpak IG and Chiralpak IK using 10% EtOH/hexane mobile phase. Better than baseline separations were obtained using 5% EtOH/hexane mobile phase on both Chiralpak IG and Chiralpak IC. Separations on Chiralpak IG, Chiralpak IK, and Chiralpak IC were studied further using EtOH/hexane mobile phase.

***Prothiophos (Compound 3)*** No separations were found for this compound using alkane/alcohol mobile phases. The intent of this study was to limit the lower level of alcohol in the mobile phase to ≥ 3.5%, so no additional conditions were attempted.

***Fenamiphos (Compound 4)*** Baseline separation or better was found on Chiralpak IA using 5% and 10% EtOH/hexane and 5% and 10% IPA/hexane, Chiralpak IC using 10% EtOH/hexane and 10% IPA/hexane, Chiralpak ID and Chiralpak IH using 5% EtOH/hexane and 5% IPA/hexane, and Chiralpak IE and Chiralpak IF using 5% EtOH/hexane. Further investigation was performed on Chiralpak IG, Chiralpak IA, and Chiralpak IC using EtOH/hexane mobile phases.

***Methamidophos (Compound 5)*** Good separations (i.e., baseline separation, or better) were found on Chiralpak IB‐N, Chiralpak IC, and Chiralpak IK. Good separation was also found on Chiralpak IH, but the peaks were broader than on the other columns listed. Peak widths are wider and retention time longer using IPA/hexane mobile phase than using EtOH/hexane mobile phase at ambient temperature on all these columns. The effect of mobile phase strength and temperature on the separation was further investigated.

***Fensulfothion (Compound 6)*** Very good separation was found on Chiralpak IH, and good separation was found on Chiralpak IA using EtOH/hexane mobile phase. On both of these columns, retention, peak width, selectivity, and resolution decrease with increase in temperature.

**FIGURE 2:**
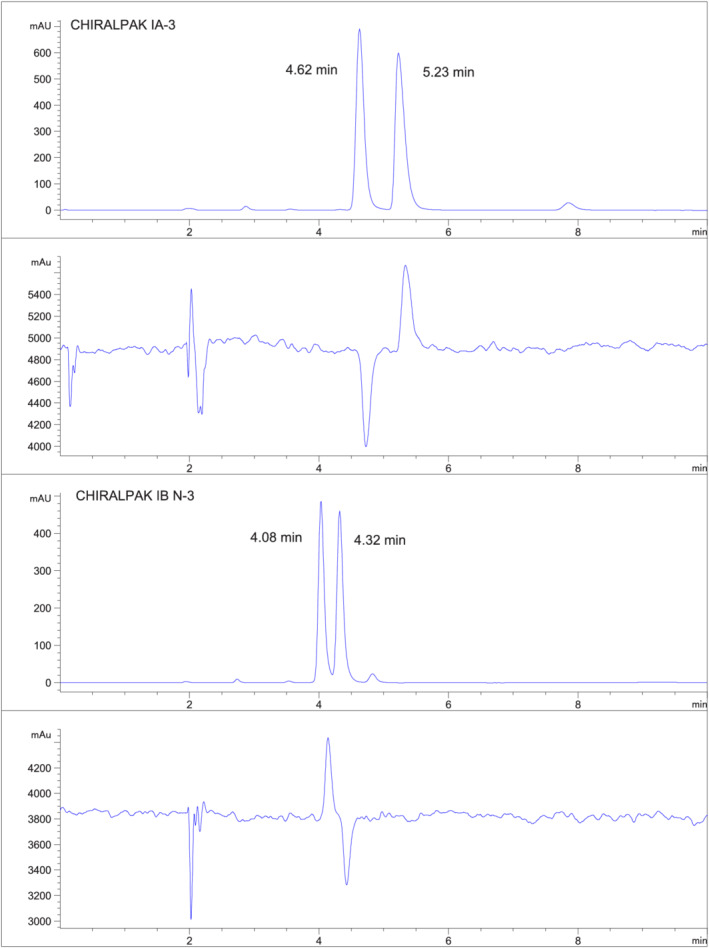
The separation of Crufomate on Chiralpak IA‐3 with 10% EtOH/90% hexane (top), the PDR‐chiral signal for Chiralpak IA‐3 (second from the top); the separation of Crufomate on Chiralpak IB‐N‐3 with 10% EtOH/90% hexane (second from the bottom), the PDR‐chiral signal for Chiralpak IB‐N‐3 (bottom)

***Crufomate (Compound 7)*** Better than baseline separations were found on Chiralpak IA and Chiralpak IG using 10%EtOH/hexane mobile phase. Baseline separations were found on Chiralpak IC and Chiralpak IH using 10% EtOH/hexane mobile phase. Less than baseline separation was found with Chiralpak IB‐N. Opposite order of elution had been found for crufomate on the coated phases, Chiralpak AD and Chiralcel OD.^3^ Chiralpak IA and Chiralpak IB‐N are the immobilized phase equivalents of Chiralpak AD and Chiralcel OD, respectively. The same order of elution was found on Chiralpak IA and Chiralpak AD, and the same order of elution was found on Chiralpak IB‐N and Chiralpak OD (Figure 2).

### 3.2 Optimization

Defining optimum performance can be subjective. “Optimum separation” depends in part on the intended needs and use of the analysis and it can depend on acceptance of trade‐offs.^33^, ^34^, ^35^ No attempt was made to “optimize” these separations for a particular application in this work, but for simplicity, they will be referred to in this paper as “optimization experiments.” Rather, the effects of temperature and mobile phase composition were investigated in this work to better understand what trade‐offs could be made to improve selectivity or resolution and to understand the sensitivity of the separation to changes in those chromatographic parameters.

Resolution increases with increasing selectivity (α) and retention factor (k) and decreasing peak width (w).^29^, ^30^, ^32^ Rather than investigating resolution directly, the parameters that affect resolution were investigated. Note that beyond affecting resolution, peak width is also often important in chiral chromatography since it also affects sensitivity (i.e., sharper peaks are taller). Obtaining sharper peaks to achieve greater sensitivity may also be an important objective of method development.

It is well established, but not always well appreciated, that method optimization by varying one‐factor‐at‐a‐time (i.e., “univariate search“) may not be very successful.^33^, ^36^, ^37^ In a systematic approach, the results of the experiment can be used to better understand how changes to the separation conditions (e.g., mobile phase strength and temperature) will affect the separation (e.g., retention time and selectivity).^33^ For example, at lower temperature, selectivity may be improved, but retention and peak width may also increase, so the optimum combination of mobile phase strength and temperature may be a trade‐off of those parameters. As a consequence, combinations of conditions may give similar chromatographic behavior. Even though initially there may appear to be more experiments, there are often fewer experiments overall.

**FIGURE 3:**
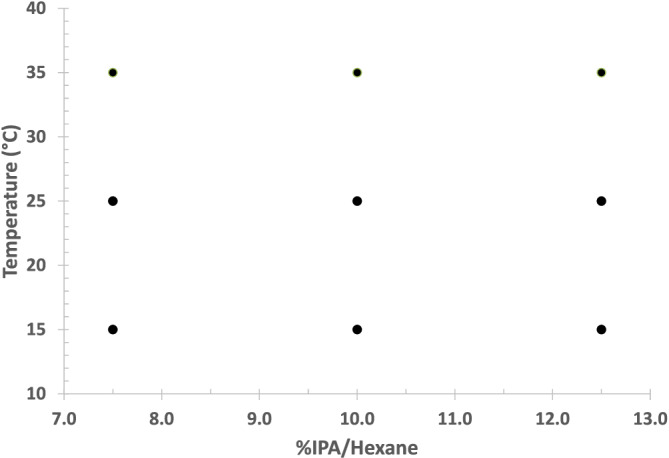
Example of a two‐factoral 3 × 3 factorial design two factors—temperature and mobile phase strength—each at three levels

The effect of varying mobile phase or temperature on retention is predictable qualitatively. The effects of varying mobile phase or temperature on selectivity and on the quality of the separation are not readily predictable and must be determined experimentally. For optimization, a simple 3 × 3 factorial design (see Figure 3) was used to investigate the effect of temperature and mobile phase strength on the separation over a typical range of combinations of temperature and mobile phase strength. Temperature was investigated at 15°C, 25°C, and 35°C. Typically, mobile phase of 10% alcohol/90% hexane was used as midlevel for the mobile phase composition. In the example design shown in Figure 3, the center point of the experimental design (10% IPA/hexane, 25°C) is similar to the screening conditions of 10% IPA/hexane and room temperature. As a further advantage of using a factorial design for optimization, the experimental results (e.g., k or α) can be presented as a family of curves. Figure 11A, as an example, presents a family of curves that show the effect of temperature on retention at different mobile phase strength. Note that the curves are parallel indicating that the temperature and mobile phase affect retention independent of each other. Also note the dashed lines indicating mobile phase/temperature combinations that will give similar retention.

Interesting results were obtained in the investigation of the combined effect of temperature and mobile phase strength using different columns on the separation of some of these compounds. For all, as expected, increasing temperature decreased retention time and decreased peak width, and increasing mobile phase strength decreased retention. Several different scenarios were observed when temperature was varied: (1) decreasing temperature improved resolution because the effect of decreasing temperature improved the selectivity more than it decreased column efficiency, (2) increasing temperture improved resolution because column efficency improved and there was little or no effect of temperature on selectivity (because of multiple interactions, as discussed further below in Section 3.2.1), and (3) varying temperature had little effect on resolution because the change in selectivity was offset by the change in column efficiency. More specific details of the separations observed are discussed below for each compound.

Full “optimization” results can be found in Table 2.

**TABLE 2:** Example mobile phase, temperature combinations that provide good separation

Cmp	Column	MP	%ROH	Temp (°C)	Sepn	tR‐1	tR‐2	k1	k2	*α*	Rs
**1**	IC	IPA	5.0	25	>B	7.93	9.27	3.96	4.79	1.21	2.6
**1**	IC	IPA	5.0	35	>B	7.02	8.17	3.39	4.11	1.21	2.6
**2**	IC	EtOH	5.0	25	>B	5.94	6.68	2.71	3.18	1.17	2.7
**2**	IG	EtOH	5.0	25	>B	6.32	7.04	2.95	3.40	1.15	5.1
**4**	IA	EtOH	10.0	25	>B	3.91	4.37	1.44	1.73	1.20	3.3
**4**	IG	EtOH	10.0	25	>B	5.76	7.03	2.60	3.39	1.31	4.7
**5**	IK	EtOH	10.0	35	>B	9.78	11.47	5.12	6.17	1.21	4.2
**5**	IK	EtOH	15.0	25	>B	6.67	7.76	3.17	3.85	1.21	3.8
**6**	IA	EtOH	10.0	25	>B	7.68	8.88	3.80	4.55	1.20	3.2
**6**	IA	EtOH	12.5	25	>B	6.24	7.04	2.90	3.40	1.17	3.0
**6**	IH	EtOH	10.0	25	>B	14.63	18.16	8.14	10.35	1.27	6.3
**6**	IH	EtOH	10.0	35	>B	11.94	14.45	6.46	8.03	1.24	5.7
**7**	IA	EtOH	10.0	25	>B	4.36	4.92	1.73	2.07	1.20	3.0

*Note*: *α*, selectivity, k2/k1. Rs, resolution, (tR‐1 ‐ tR‐2)/([width‐1 + width‐2]/2), width projected to 4σ, computed by ChemStation. >B, peak separation better than baseline resolution. MP, mobile phase, alcohol and hexane. Cmp, compound, number as shown in Figure 1.

**FIGURE 4:**
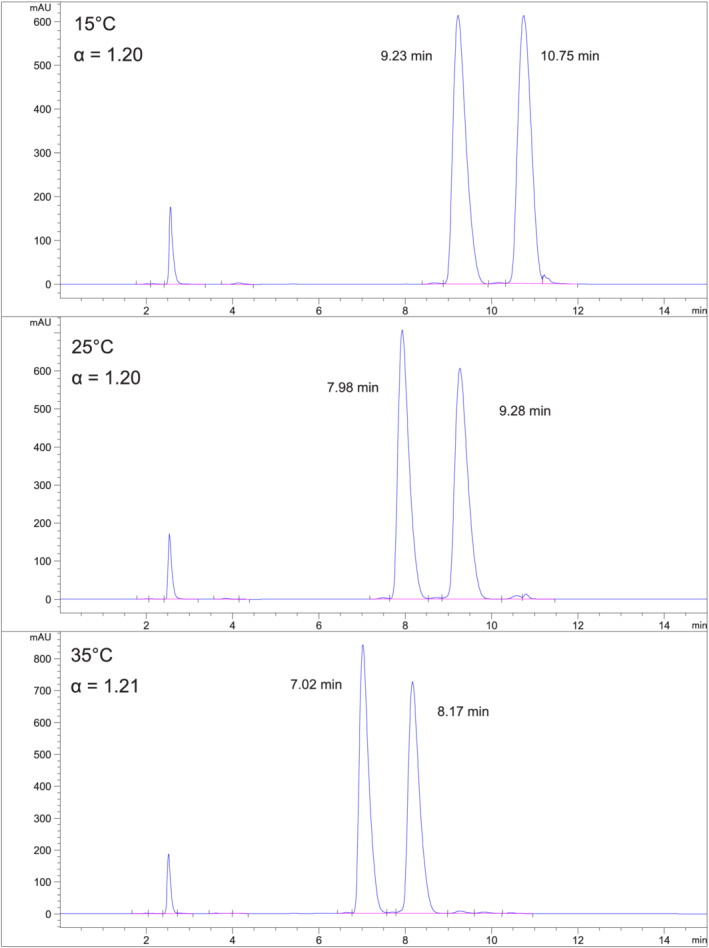
Effect of varying temperature at 5% IPA/hexane on selectivity and separation of profenofos on Chiralpak IC

***Profenofos (Compound 1) on Chiralpak IC*.** The additional study to improve the separation was limited to using Chiralpak IC with IPA/hexane mobile phase for this compound. The separation on Chiralpak IC is an example of a contest between selectivity and peak sharpness to improve resolution. At 10% IPA, selectivity decreases and peak sharpness increases with increasing temperature. Because of the broadness of the peaks at 15°C, the peaks were less than baseline resolved. At 25°C, the peaks were sharper and baseline separated. At 35°C, the peaks are sharper but closer together, so the peaks are less than baseline resolved. As shown in Figure 4, at 5% IPA/hexane, there is a slight decrease in selectivity between 15°C and 35°C. However, by inspection, resolution is equivalent because of decreasing peak width with increasing temperature.

***Malathion (Compound 2) on Chiralpak IC***. The separation on Chiralpak IC using EtOH/hexanes mobile phase was further investigated at 5%, 7.5%, and 10% EtOH/hexanes mobile phase. In this separation, the effect of temperature on selectivity is more important than the effect of temperature on peak width. For example, better than baseline separation is obtained at 15°C and 25°C; but only baseline separation is obtained at 35°C.

***Malathion (Compound 2) on Chiralpak IG***. On Chiralpak IG, effect of increased selectivity at lower temperature was more important than decreased peak width at higher temperature. Using 5% EtOH/hexane mobile phase at 15°C k‐1 was < 5 and the enantiomer peaks were better than baseline resolved.

***Malathion (Compound 2) on Chiralpak IK*.** The separation on Chiralpak IK using EtOH/hexanes mobile phase was further investigated at 7.5%, 10%, and 12.5% EtOH/hexanes mobile phase. Better selectivity was found at lower temperature. Slight improvement was found at weaker mobile phase strength. At 7.5% EtOH/hexane mobile phase, greater than baseline separation was obtained at 15°C, baseline separation was obtained at 25°C and less than baseline separation was obtained at 35°C. At 10% EtOH/hexane, baseline separation was obtained at 15°C and less than baseline separation was obtained at 25°C.

***Fenamiphos (Compound 4) on Chiralpak IA***. Better than baseline separation was obtained on Chiralpak IA using hexane/EtOH mobile phase at 15°C, 25°C, and 35°C. The separation was initially evaluated using 7.5%, 10%, and 12.5% EtOH/hexane mobile phase. It was observed that there was apparent random behavior of selectivity at different mobile phase/temperature combinations. Additional experiments on Chiralpak IA were performed using 5 × 5 factorial design. Two additional temperature levels, 20°C and 30°C, and two additional mobile phase levels, 8.8% and 11.3% EtOH/hexane, were added to the 3 × 3 factorial design.

**FIGURE 5:**
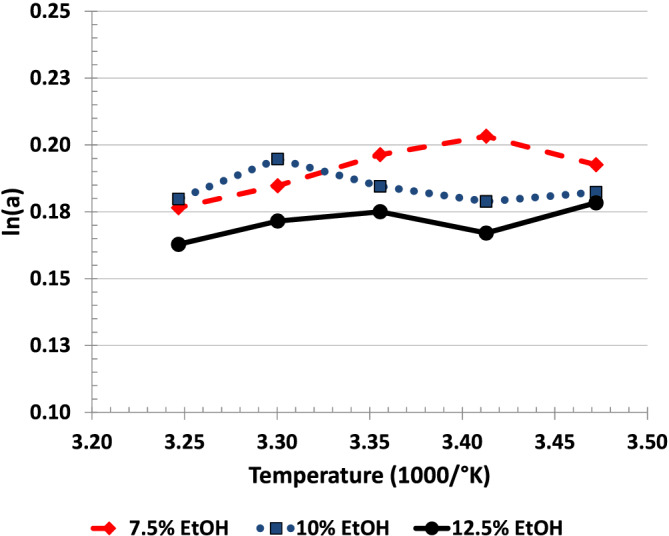
Vant Hoff plot showing limited effect of temperature on selectivity for Fenamiphos on Chiralpak IA using EtOH/hexane mobile phase. Results were obtained using 5 × 5 factorial design; for better clarity, only three mobile phase levels (7.5% EtOH [blue], 10% EtOH [red], and 12.5% EtOH [green]) are shown

Figure 5 shows the Van't Hoff plot for selectivity. The results of the 8.8% and 11.3% EtOH/hexane mobile phase experiments are not shown for clarity. Figure 7 shows that there is a mobile phase strength effect on selectivity (generally better selectivity with weaker mobile phase) but no systematic temperature effect.

**FIGURE 6:**
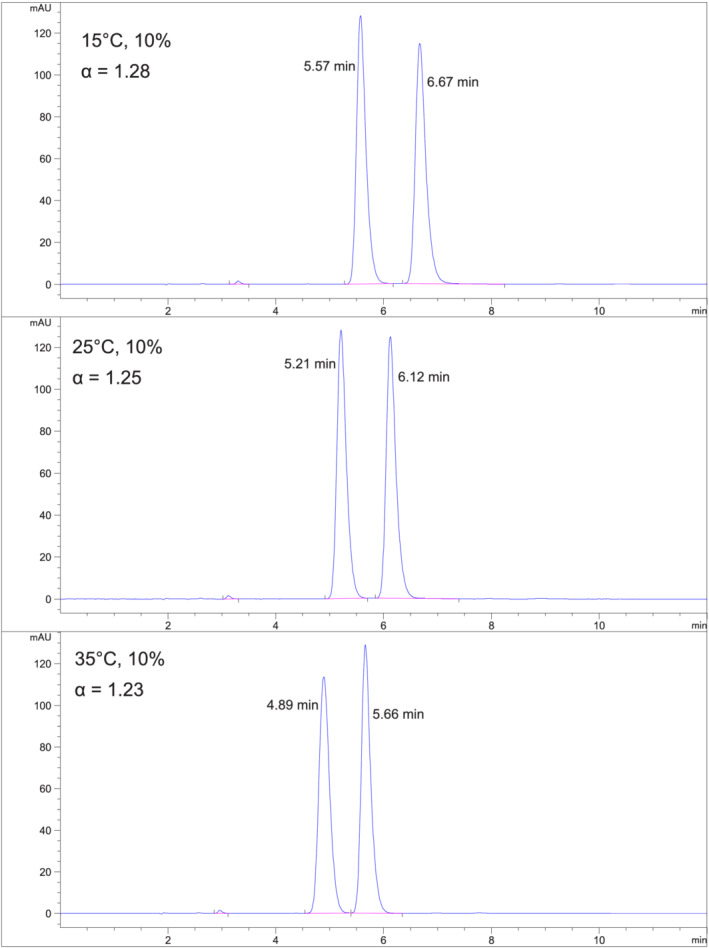
Separation of Fenamiphos on Chiralpak IC using 10% EtOH/hexane mobile phase. For this separation temperature has only a slight effect on peak width and retention. Selectivity decreases with increasing temperature

***Fenamiphos (Compound 4) on Chiralpak IC***. The 10%, 15%, and 20% EtOH/hexane mobile phases were evaluated on Chiralpak IC. Selectivity decreased with increasing temperature at constant mobile phase strength on Chiralpak IC. There is little effect of mobile phase strength on selectivity. Acceptable retention (k1 ~ 2.3) and good separation was obtained at 10% EtOH/hexanes at 25°C (Figure 6).

***Fenamiphos (Compound 4) on Chiralpak IG***. Good separation was obtained at 5%, 7.5%, and 10% EtOH/hexane mobile phase levels at the temperatures investigated (i.e., 15°C, 25°C, and 35°C) on Chiralpak IG. Resolution decreased with increasing temperature since the decrease in peak width is not as important as the decrease in selectivity with increasing temperature.

***Methamidophos (Compound 5) on Chiralpak IK***. Using EtOH/hexane mobile phase on Chiralpak IK, the effect of temperature on retention and selectivity is well described by the van't Hoff equation. Methamidophos is well retained on this column, so mobile phase of 10% to 15% EtOH/hexane would be used to obtain a retention factor of ~ 3 to 5 for the first eluting enantiomer. Because of the good separation, temperatures such as 25°C to 35°C can be used to obtain sharper peaks.

**FIGURE 7:**
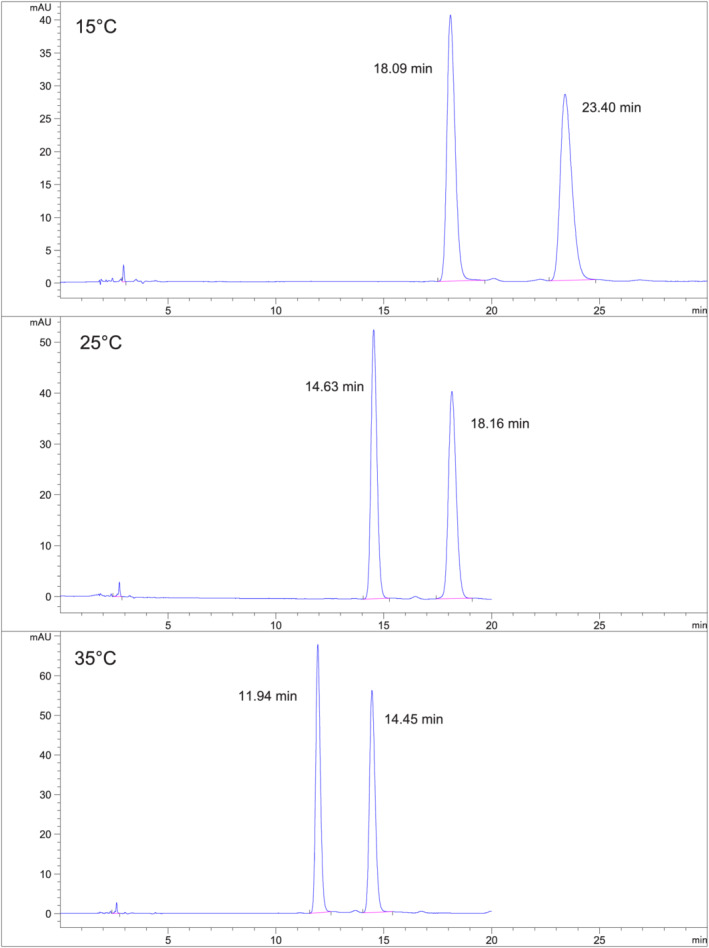
Separation of fensulfothion on Chiralpak IH at 15°C, 25°C, and 35°C using 10% EtOH/hexane mobile phase

***Fensulfothion (Compound 6) on Chiralpak IH***. Figure 7 shows chromatograms at 15°C, 25°C, and 35°C using 10% EtOH/hexane on Chiralpak IH. Separation is much greater than baseline resolution. About 25°C (or higher) can be used for this separation to allow shorter retention times and sharper peaks.

**FIGURE 8:**
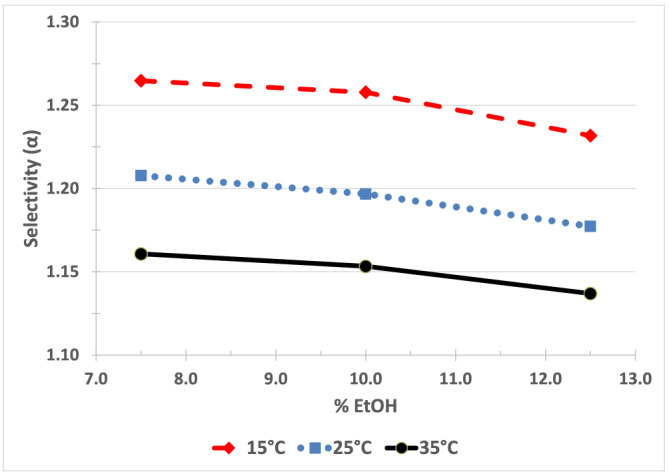
Effect of %EtOH/hexane mobile phase on selectivity for fensulfothion on Chiralpak IA. The effect of temperature on selectivity for fensulfothion on Chiralpak IA is shown in Figure 11D

***Fensulfothion (Compound 6) on Chiralpak IA***. Figure 8 shows that for fensulfothion, there is only a limited effect of mobile phase strength on selectivity using hexane/EtOH mobile phase on Chiralpak IA. Figure 11D shows the effect of temperature on selectivity using 10% EtOH/hexanes mobile phase. From an optimization standpoint, this would indicate that lower temperature could be used to increase selectivity and mobile phase strength could be used to control retention without greatly decreasing selectivity.

**FIGURE 9:**
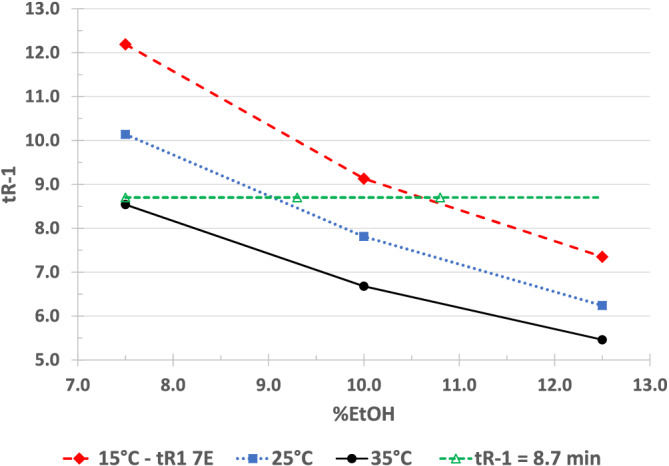
Effect of mobile phase on retention of fensulfothion on Chiralpak IA. Dashed and dotted lines show combinations of mp and temperature that would be expected to give similar retention

**FIGURE 10:**
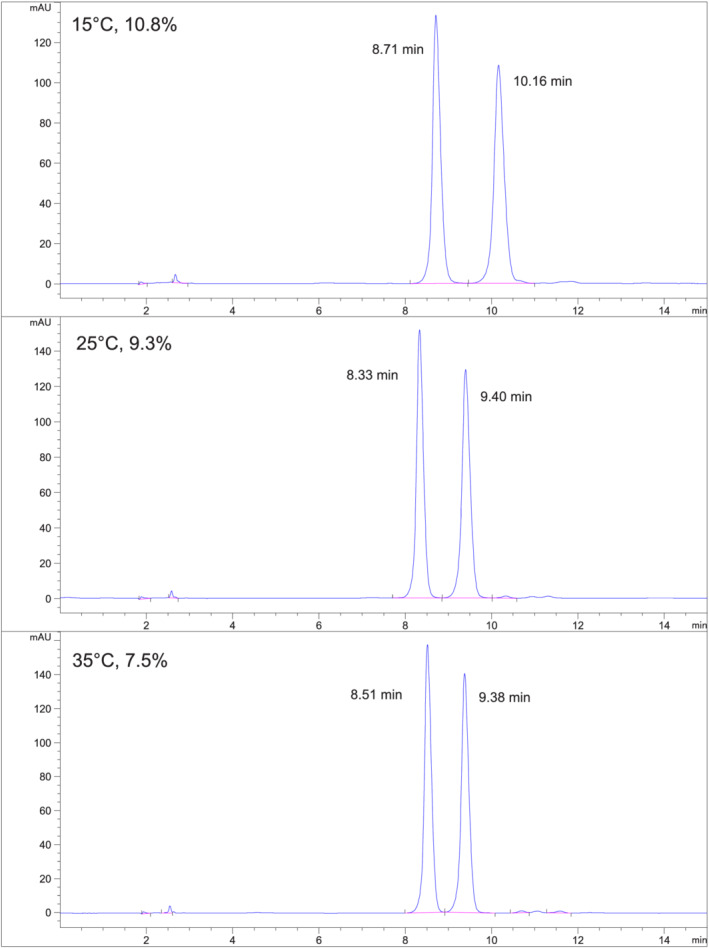
Combinations of mobile phase (EtOH/hexane) and temperature giving similar retention of fensulfothion on Chiralpak IA as predicted in Figure 9

This separation is the example shown in Figure 9 of predicting retention time for a given combination of mobile phase and temperature. Figure 10 shows chromatograms obtained at (35°C, 7.5% EtOH), (25°C, 9.3% EtOH), and (15°C, 10.8% EtOH).

The retention time of the first eluting enantiomer is nearly the same in each chromatogram as predicted from Figure 9. The selectivity is better at lower temperature as would be predicted from Figure. The nine experiments in the 3 × 3 factorial design provide a useful means of predicting the combination of mobile phase strength and temperature to achieve the desired retention and selectivity from a small number of systematic experiments.

#### 3.2.1 Further discussion on the effect of temperature

The investigation into the effects of varying temperature on retention, peak width, and selectivity, as factors that determine resolution obtained for these compounds, had two objectives: (1) improve separation and (2) determine the sensitivity of the separations to changes in chromatographic conditions. In some cases, varying temperature had an important effect on selectivity; in other cases, temperature had little effect on selectivity.

**FIGURE 11:**
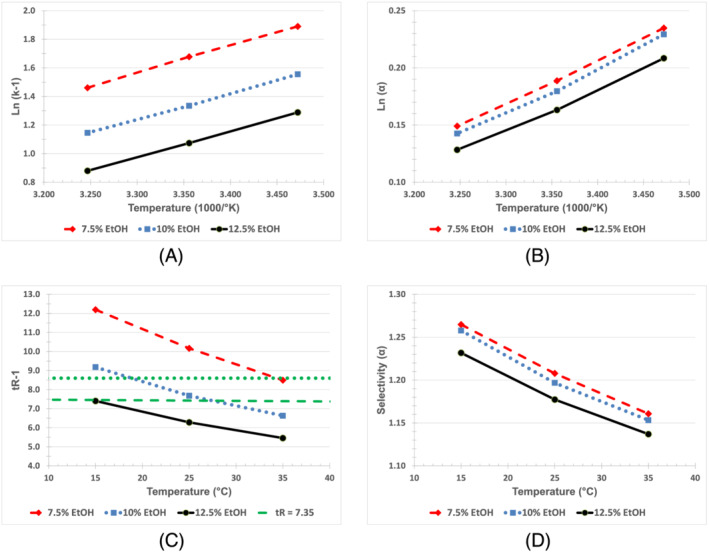
(A) Vant Hoff plot of retention of first enantiomer of fensulfothion on Chiralpak IA. Dashed line indicates mp/temperature combinations that would be expected to give similar retention of 7.35 min (k 3.4). (B) Vant Hoff plot of selectivity of fensulfothion on Chiralpak IA. Note that parallel curves indicate that temperature and mp effect selectivity independently (i.e., no interaction between the factors). (C) Effect of temperature on retention of fensulfothion on Chiralpak IA. Compare with Vant Hoff plot in Figure 11A. Dashed and dotted lines indicate mp/temperature combinations that would be expected to give similar retention of 7.35 and 8.6 min, respectively (t_R‐1_ is retention time, in minutes, of first eluting enantiomer). (D) Effect of temperature on selectivity of fensulfothion on Chiralpak IA. Compare with Vant Hoff plot in Figure 11B. Shown are 7.5% EtOH (blue), 10% EtOH (red), and 12.5% EtOH (green)

The effect of temperature on selectivity and retention is often described quantitatively as an equilibrium process using the van't Hoff equation (Figure 11A,B). However, note that the effect of temperature on selectivity as described by the van't Hoff equation may not adequately describe selectivity in situations, such as enantio‐selective chromatography that may have more than one dominate interaction between stationary phase, mobile phase, and analyte.^38^, ^39^, ^40^ This means that in some chiral separations, temperature may have little apparent (or overall) effect on selectivity. This could be unfortunate since temperature is not available as a parameter that can be varied to improve selectivity. However, this could actually be beneficial in cases where the mobile phase and CSP provide good selectivity and increased temperature can be used to increase the sharpness of the peaks with little or no loss in selectivity.

The objective of this work was to determine the effect of temperature on the separation rather than to investigate the thermodynamics involved. Ideally, the data should be able to be presented so that the relationship can be understood intuitively. For this reason, several of the plots of the effect of temperature on selectivity or retention are presented as *α* (or k or t_R_) versus °C, which is often easier to interpret than the van't Hoff plots (compare Figure 11A–C and Figure 11B–D for instance). Note that over a small temperature range (e.g., 15°C to 35°C), the plot of 1/°K versus °C is nearly a straight line with a negative slope. (The correlation coefficient for a first‐order regression line generated using independent variable of 15°C, 20°C, 25°C, 30°C, and 35°C is >0.99. Note also that for a small range for selectivity such as 1.0 to 1.5, ln(x) versus x is also nearly a straight line. As an example using six values of x from 1.0 to 1.5 in 0.1 increments, the line of ln(x) versus x is also nearly straight. The correlation coefficient for this first order regression line is also > 0.99.).

## 4 CONCLUSION

Representatives of the OPs that had been previously separated on the coated polysaccharide CSPs were successfully separated on a several new immobilized polysaccharide CSPs. In a number of cases, good (i.e., greater than baseline) separations were found on multiple columns, representing new separations not previously reported in the literature (e.g., the separation of Methamidophos on Chiralpak IK and the separation of Fensulfothion on Chiralpak IH).

A number of interesting results were also obtained related to the interplay between temperature, mobile phase strength, and retention. In all cases, increasing temperature or increasing mobile phase strength decreased retention time. But in some cases, changing temperature and/or mobile phase strength did not affect selectivity. For example, for profenofos on Chiralpak IC, malathion on Chiralpak IG, and fenamiphos on Chiralpak IA, it was observed that temperature had little effect on selectivity. For malathion on Chiralpak IG, fenamiphos on Chiralpak IA, and for fensulfothion on Chiralpak IA, it was observed that mobile phase strength had little effect on selectivity.

Using column switching and a systematic approach, separations were found in easily automated, overnight runs, and an understanding was obtained into what changes could be made if changes to the separation were desired. It was also demonstrated that it is possible to predict what trade‐offs could be made in changing a separation. Collecting information in a systematic way simplified the understanding of potentially confusing systems, such as the separations where changes in mobile phase and/or temperature had little effect on selectivity. Or as in some cases, poor peak shape that was observed at only one set of conditions. Since the data were collected as a set, it was possible to determine that the poor shape occurred over a limited range of mobile phase/temperature combinations. In a “random walk” design, it is likely that these effects would have been very confusing.

## Data Availability

The authors confirm that the data supporting the findings of this study are available within the article or its supporting information.
